# Adolescents with type 1 diabetes can achieve glycemic targets on intensive insulin therapy without excessive weight gain

**DOI:** 10.1002/edm2.352

**Published:** 2022-06-17

**Authors:** Alexandra L. Marlow, Bruce R. King, Helen T. Phelan, Carmel E. Smart

**Affiliations:** ^1^ School of Medicine and Public Health University of Newcastle Callaghan New South Wales Australia; ^2^ Hunter Medical Research Institute New Lambton Heights New South Wales Australia; ^3^ Department of Pediatric Endocrinology and Diabetes John Hunter Children's Hospital New Lambton Heights New South Wales Australia

**Keywords:** adolescents, body mass index, glycemic control, type 1 diabetes

## Abstract

**Introduction:**

The aim of this study was to compare glycemic control and body mass index standard deviation score (BMI‐SDS) before and after implementation of intensive insulin therapy using multiple daily injection (MDI) or continuous subcutaneous insulin infusion (CSII) in adolescents with type 1 diabetes (T1D) attending a large multidisciplinary paediatric diabetes clinic in Australia.

**Methods:**

Prospective data were collected for cross‐sectional comparison of youth aged 10.0–17.9 years (*n* = 669) from routine follow‐up visits to the diabetes clinic in 2004, 2010, and 2016. Outcome measures included HbA1c; BMI‐SDS; and insulin regimen.

**Results:**

BMI‐SDS remained stable between 2004 to 2016 in the 10–13 and 14–17 year age group (0.7 vs. 0.5, *p* = .12 and 0.7 vs. 0.7, *p* = .93, respectively). BMI‐SDS was not different across HbA1c groups; <53 mmol/mol (7.0%), 53 to <75 mmol/mol (<7.0 to <9.0%) and >75 mmol/mol (>9.0%) in 2004 (*p* = .873), 2010 (*p* = .10) or 2016 (*p* = .630). Mean HbA1c decreased from 2004 to 2016 in the 10–13 year (69 mmol/mol (8.4%) vs. 57 mmol/mol (7.4%), *p* = <.001) and 14–17 year group (72 mmol/mol (8.7%) vs. 63 mmol/mol (7.9%), *p* = <.001). Prior to the implementation of MDI and CSII in 2004 only 10% of 10–13 year olds and 8% of 14–17 year olds achieved the international target for glycemic control (HbA_1c_ 53 mmol/mol [<7.0%]). In 2016, this increased to 31% of 10–13 year olds and 21% of 14–17 year olds.

**Conclusions:**

BMI‐SDS did not increase with the change to intensive insulin therapy despite a doubling in the number of adolescents achieving the recommended glycemic target of <7.0% (53 mmol/mol). HbA1c was not associated with weight gain.

## Graphical abstract

The aim of this study was to compare glycemic control and body mass index standard deviation score (BMI‐SDS) before and after implementation of intensive insulin therapy in adolescents with T1D. Prospective data were collected for cross‐sectional comparison of youth aged 10.0‐17.9 years (n=669) from routine follow‐up visits to the diabetes clinic in 2004, 2010 and 2016. BMI‐SDS did not increase with the change to intensive insulin therapy despite a doubling in the number of adolescents achieving the recommended glycemic target of 7.0% (53mmol/mol).

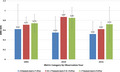

## 1 INTRODUCTION

High rates of overweight and obesity are shown across paediatric type 1 diabetes (T1D) cohorts, including 32% from the international SWEET registry^1^ and 33% from the Australasian Diabetes Data Network registry.^2^ A greater body mass index standard deviation score (BMI‐SDS) was observed in youth from the Diabetes Prospective Follow‐up cohort compared with a national reference value (0.65 vs. 0.33).^3^ Similarly, our clinic identified rates of overweight and obesity in T1D exceeding those of individuals in the general Australian population (37% vs. 23%).^4^

It was shown in the Diabetes Control and Complications Trial that adolescents and adults on intensive insulin therapy gain significantly more weight, with an average increase of 4 kg more than those following conventional therapy.^5^ Until the end of 2004, individuals attending the John Hunter Children's Hospital (JHCH) paediatric diabetes clinic were mostly using conventional twice daily insulin injections. In 2005, insulin treatment changed to flexible multiple daily injection (MDI) therapy or continuous subcutaneous insulin infusion (CSII). Family education focused on normalizing glycemia to meet international targets^6^ including rapid responses to increases in glycated haemoglobin (HbA1c) with insulin adjustments and intensive re‐education.

The nutrition education principles, including appropriate individualized energy and percent macronutrient intakes, were the same across the analysed period from 2004 to 2016, and were guided by the International Society for Paediatric and Adolescent Diabetes (ISPAD) Clinical Practice Consensus Guidelines.^7^ Emphasis was placed on avoidance of continuous grazing, family‐based meal‐time routines, dietary quality and maintenance of a healthy body weight.^7^ The introduction of insulin to carbohydrate ratios across the whole clinic enabled greater flexibility in carbohydrate intake at meals and snacks^8^ although upper limits of carbohydrate intake were still recommended for age. Individuals attended 3‐monthly outpatient visits across the entire study period.

The aim of this study was to compare BMI‐SDS and glycemic control before and after implementation of intensive insulin therapy in adolescents with T1D.

## 2 RESEARCH DESIGN AND METHODS

Ethics approval was granted by the Hunter New England Human Research Ethics Committee (Reference, 08/11/19/5.04). Inclusion criteria were T1D diagnosis and aged 10.0–17.9 years at time of visit. Routine clinical data including age, sex, diabetes duration, HbA1c, height, weight and insulin regimen were collected prospectively during 3‐monthly outpatient appointments. Data was extracted from 2004 prior to the commencement of intensive insulin therapy and compared to data from 2010 and 2016. Continuous outcome variables were averaged for each individual over 12‐month time periods. The year 2020 was not included due to possible effects of the COVID‐19 pandemic. HbA_1c_ was measured using the DCA‐Vantage. Height was measured using calibrated Harpenden stadiometer and weight using calibrated scales. BMI‐SDS and International Obesity Taskforce Guidelines (IOTF) classification are reported.^9^ Insulin regimens were twice daily injection (BD), fixed MDI (three fixed quick acting and one long acting injection per day), flexible MDI (insulin dose calculated using an insulin to carbohydrate ratio and sensitivity factor)^8^ or CSII, with the regimen last recorded during the year included in the analysis.

Statistical analyses were performed using STATA I/C version 15.0 (StataCorp LLC).^10^ Descriptive statistics reported as mean ± standard deviation (SD) for continuous variables and frequency and percentage (%) for categorical variables. To determine statistical significance of continuous variables, we used one‐way analysis of variance for normally distributed data and Wilcoxon rank test (Mann–Whitney) for non‐parametric data, and Fisher's exact test for categorical variables. Tests were performed as two‐sided analysis with a level of <0.05 considered significant.

## 3 RESULTS

The characteristics of 669 eligible youth are summarized in Table 1. In 2004, patterns of insulin use were BD 60% (*n* = 130), fixed MDI 28% (*n* = 62) and CSII 12% (*n* = 26). By 2016, there was minimal uptake of BD therapy (<1%, *n* = 1), with almost equal parts using flexible MDI (52%, *n* = 119) and CSII (47%, *n* = 108) therapy. BMI‐SDS remained stable between 2004 and 2010 in both the 10–13 and 14–17 year age groups (0.7 vs. 0.8, *p* = .93 and 0.7 vs. 0.9, *p* = .13, respectively), and again in 2004 to 2016, in the 10–13 and 14–17 year age group (0.7 vs. 0.5, *p* = .12 and 0.7 vs. 0.7, *p* = .93, respectively). BMI‐SDS also remained stable between 2010 and 2016 in both the 10–13 and 14–17 year age groups (0.8 vs. 0.5, *p* = .083 and 0.9 vs. 0.7, *p* = .262, respectively). In 2004, 39% (*n* = 40) of 10–13 year olds were overweight (31%, *n* = 32) or obese (8%, *n* = 8), whereas in 2016, 29% (*n* = 30) were overweight (24%, *n* = 24) or obese (6%, *n* = 6); however, this difference was not significant (*p* = .18). Rates of overweight or obesity remained stable in the 14–17 year age group. In 2004, 40% (*n* = 46) of 14–17 year olds were overweight (26%, *n* = 30) or obese (14%, *n* = 16), and in 2016, 40% (*n* = 51) were overweight (29%, *n* = 37) or obese (11%, *n* = 14; *p* = 1.0).

**TABLE 1:** Clinical characteristics of 669 youth aged 10–13 years or 14–17 years with type 1 diabetes by year of visit

Year	2004		2010		2016		2004 versus 2010 (p)		2004 versus 2016 (p)	
Age group (years)	10–13	14–17	10–13	14–17	10–13	14–17	10–13	14–17	10–13	14–17
Number of children	102	116	98	125	102	126				
Age (years)	12.1 (1.5)	15.8 (1.1)	12.1 (1.2)	15.9 (1.2)	12.0 (1.1)	16.0 (1.1)	0.977	0.524	0.529	0.272
Sex (male)	48 (47)	67 (58)	43 (44)	75 (60)	48 (47)	70 (56)	0.672	0.794	1.000	0.795
Diabetes duration (years)	4.6 (3.6)	5.8 (4.0)	4.9 (3.2)	6.9 (3.9)	4.9 (3.2)	6.6 (4.3)	0.292	0.025	0.367	0.157
HbA1c mean (NGSP)	8.4 (1.2)	8.7 (1.5)	7.9 (1.2)	8.3 (1.4)	7.4 (1.0)	7.9 (1.5)	0.009	0.022	<0.001	<0.001
HbA1c mean (IFCC)	69 (13)	72 (16)	64 (13)	67 (15)	57 (10)	63 (17)				
HbA1c Category NGSP (IFCC)										
<6.5 (48)	6 (6)	5 (4)	10 (10)	10 (8)	15 (15)	14 (11)				
≥6.5 < 7.0 (≥48 < 53)	4 (4)	4 (3)	7 (7)	10 (8)	17 (17)	13 (10)				
≥7.0 < 7.5 (>53 < 58)	9 (9)	11 (9)	14 (14)	17 (14)	24 (24)	32 (25)				
≥7.5 < 9.0 (≥58 < 75)	53 (52)	55 (47)	54 (54)	55 (44)	41 (40)	45 (36)				
≥9.0 (≥75)	30 (29)	41 (35)	13 (13)	33 (26)	5 (5)	22 (17)				
HbA1c <6.5 (48)^a^	6 (6)	5 (4)	10 (10)	10 (8)	15 (15)	14 (11)	0.304	0.294	0.063	0.058
HbA1c <7.0% (53)^b^	10 (10)	9 (8)	17 (17)	20 (16)	32 (31)	27 (21)	0.049	0.033	<0.001	<0.001
HbA1c <7.5 (58)^c^	19 (19)	20 (17)	31 (32)	37 (30)	56 (55)	59 (47)	0.148	0.073	0.000	0.003
BMI‐SDS, mean (CDC)	0.7 (0.9)	0.7 (0.9)	0.8(0.8)	0.9 (0.8)	0.5 (0.9)	0.7 (0.9)	0.934	0.126	0.115	0.930
Weight category										
Underweight^d^	2 (2)	3 (3)	0 (0)	2 (2)	5 (5)	4 (3)	0.498	0.674	0.445	1.000
Normal^d^	60 (59)	67 (58)	61 (62)	65 (52)	67 (66)	71 (56)	0.666	0.437	0.386	0.897
Overweight^d^	32 (31)	30 (26)	28 (29)	41 (33)	24 (24)	37 (29)	0.758	0.260	0.272	0.568
Obese^d^	8 (8)	16 (14)	9 (9)	17 (14)	6 (6)	14 (11)	0.803	1.000	0.783	0.563
Overweight or obese^d^	40 (39)	46 (40)	37 (38)	58 (46)	30 (29)	51 (40)	0.885	0.301	0.184	1.000
Insulin regimen										
BD	69 (68)	61 (53)	5 (5)	7 (6)	0 (0)	1 (1)	<0.001	<0.001	<0.001	<0.001
MDI	22 (22)^†^	40 (35)^†^	58 (59)	75 (60)	48 (47)	71 (56)	<0.001	<0.001	<0.001	0.001
CSII	11 (11)	15 (13)	35 (36)	43 (34)	54 (53)	54 (43)	<0.001	<0.001	<0.001	<0.001

*Note*: Data are *n* (%), mean ± SD.

Fixed MDI^†^ defined as three fixed quick acting and one long acting injection per day, flexible MDI defined as ≥4 injections per day, CSII continuous subcutaneous insulin infusion pump therapy.

^a^ National Institute of Health and Care Excellence (NICE) Target.^21^

^b^ 2018 ISPAD Target.^6^

^c^ 2014 ISPAD Target.^22^

^d^ Defined according to IOTF adjusted for age (2–18 years of age) and sex.^9^

**FIGURE 1:**
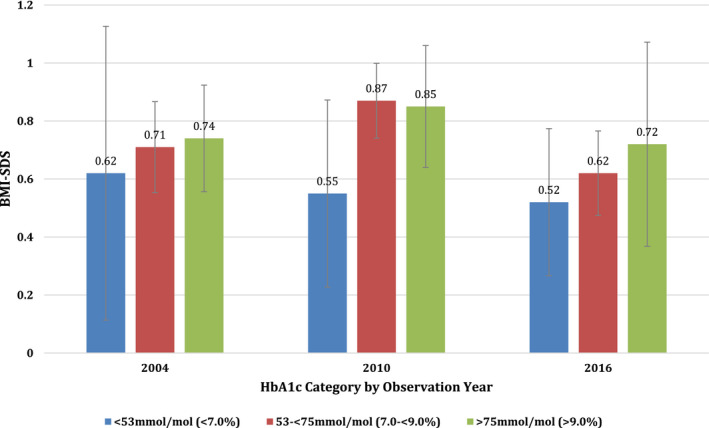
BMI‐SDS by HbA1c category over observation years

There was no significant difference in mean BMI‐SDS across HbA1c groups (<53 mmol/mol (7.0%)), 53 to <75 mmol/mol (<7.0 to <9.0%) and >75 mmol/mol (>9.0%)) in 2004 (0.62, 0.71 and 0.74, respectively (*p* = .873)), 2010 (0.55, 0.87 and 0.85, respectively (*p* = .10)) or 2016 (0.52, 0.62 and 0.72, respectively *p* = .630; see Figure 1). There was also no significant difference in mean BMI‐SDS across therapy type in 2004 (BD 0.67, fixed MDI 0.84 and CSII 0.61, *p* = .382), in 2010 (BD 1.16, flexible MDI 0.79 and CSII 0.80, *p* = .295) and in 2016 (BD 0.74, MDI 0.67 and CSII 0.54, *p* = .528).

Mean HbA1c decreased significantly from 2004 to 2010 in both the 10–13 (69 mmol/mol (8.4%) vs. 57 mmol/mol (7.9%), *p* = .005) and 14–17 year group (72 mmol/mol (8.7%) vs. 67 mmol/mol (8.3%), *p* = .02), and again from 2004 to 2016 in the 10–13 (69 mmol/mol (8.4%) vs. 57 mmol/mol (7.4%), *p* = <.001) and 14–17 year group (72 mmol/mol (8.7%) vs. 63 mmol/mol (7.9%), *p* = <.001). Mean HbA1c also decreased significantly from 2010 to 2016 in both the 10–13 (64 mmol/mol (7.9%) vs. 57 mmol/mol (7.4%), *p* = <.001) and 14–17 year group (67 mmol/mol (8.3%) vs. 63 mmol/mol (7.9%), *p* = .003). In 2016, 31% (*n* = 32) of 10–13 year olds achieved the international target HbA1c of <53 mmol/mol (<7.0%)^6^ compared to 10% (*n* = 10) in 2004 (*p* = <.001). In the 14–17 year old group, 21% (*n* = 27) had a mean HbA1c of <7.0% (53 mmol/mol) in 2016, compared to 8% (*n* = 9) in 2004 (*p* = <.001).

There was no significant difference in severe hypoglycemic events in years 2004 versus 2010 (13 vs. 14, *p* = 1.000) or years 2004 versus 2016 (13 vs. 6, *p* = .101).

## 4 DISCUSSION

This data suggests that in adolescents with T1D, it is possible to achieve target glycemic control on an intensified insulin regimen without excessive weight gain, and that there is no association between BMI‐SDS and HbA1c or therapy type. Our findings are in contrast to those from adolescents in the DCCT trial which saw a two‐fold greater risk of becoming overweight with intensive management.^11^ Contrary to other reports that demonstrate adolescents are a group that have suboptimal glycemic outcomes,^12^ our data showed a doubling (14–17 year group) and tripling (10–13 year group) of youth meeting international glycemic targets on intensive insulin therapy. Our team previously demonstrated in toddlers that mealtime structure plays an important role in achieving glycemic targets^13^ and it is possible this message in older children and adolescents may assist with maintaining weight whilst meeting glycemic outcomes.

Results of this study also indicate a decline in youth meeting international glycemic targets from the youngest (31%) to the oldest age group (21%). A decline in metabolic control during adolescence has been demonstrated internationally, with an average HbA1c increase of 8 mmol/mol (0.7%) from childhood to adolescence.^14^ Mean HbA1c in the 14–17 year group of this study (63 mmol/mol (7.9%)) is lower than current data from adolescents in England (74 mmol/mol [8.9%]), Wales (76 mmol/mol [9.1%]) and USA (73 mmol/mol [8.8%]), and comparable to results from Sweden (62 mmol/mol [7.8%]).^14^ Improved glycemic control was not at the expense of increased hypoglycemia. This is consistent with results from the Hvidore study which showed that the risk of severe hypoglycemia was lowest in paediatric diabetes centres with the tightest glycemic control.^15^

Despite almost 100% of adolescents using intensive insulin therapy by 2016, rates of overweight and obesity were unchanged from 2004–2016 (39% vs. 29% in the 10–13 year group and 40% vs. 40% in the 14–17 year group). This reflects a similar trend to the plateauing of rates of overweight and obesity among adolescents (12–17 years) in the general population.^16^ However, rates of overweight and obesity in adolescents with T1D still exceed those seen in the Australian population.^4^

The pathophysiology of excess weight gain in youth with T1D is not clear; however, multiple factors are common across adolescents with or without T1D, such as sedentary behaviour, reduced exercise (especially in females) and unhealthy eating habits which may contribute to favour a positive energy balance.^17^ Factors unique to people with T1D include fear of hypoglycemia and fear of loss of diabetes control as barriers to physical activity,^18^ and consumption of additional carbohydrates to prevent or correct hypoglycemia.^19^

It is important that centres implement a model of care that supports proactive insulin adjustment around physical activity, and meal‐time routines focusing on healthy eating habits which meet nutrient requirements and avoid excess snacking. These factors likely play a pivotal role in supporting adolescents to achieve target glycemic control without excessive weight gain.

There were several limitations in this study. A limitation is that we report from a single diabetes centre, however JHCH is a large university teaching hospital, providing care to all youth with type 1 diabetes across a wide socio‐economic background in a nationalized health scheme, in the Hunter Region of NSW, Australia. Body composition analysis was not performed so it is possible muscular individuals may be miscategorised as overweight. Data were captured across three time‐points, thus some participants may have been included at more than one time‐point. The year 2020 was not included in this analysis due to the possible COVID‐19 pandemic effects on bodyweight, with one large US population study observing a doubling of mean adjusted BMI in 10–13 year olds during the pandemic.^20^ Puberty may have impacted body weight but we did not record pubertal status.

In conclusion, the results of this study challenge the opinion that weight gain is a side effect of intensified insulin regimens and improvements in glycemic control. Adolescence remains a challenging period for individuals with T1D with declining glycemic control and a high prevalence of overweight and obesity. Further investigation into the causes of weight gain in adolescents with T1D is vital to informing future interventions.

## 5 AUTHOR CONTRIBUTIONS

**Alexandra L. Marlow:** Conceptualization (equal); data curation (equal); formal analysis (lead); investigation (equal); methodology (equal); project administration (equal); resources (equal); software (equal); validation (equal); visualization (lead); writing – original draft (lead); writing – review and editing (equal). **Bruce R. King:** Conceptualization (equal); data curation (supporting); formal analysis (supporting); investigation (equal); methodology (equal); project administration (equal); resources (equal); supervision (equal); validation (supporting); visualization (supporting); writing – original draft (supporting); writing – review and editing (supporting). **Helen T. Phelan:** Conceptualization (equal); data curation (equal); formal analysis (supporting); investigation (equal); methodology (supporting); project administration (supporting); writing – original draft (supporting); writing – review and editing (supporting). **Carmel E. Smart:** Conceptualization (equal); data curation (supporting); formal analysis (supporting); investigation (equal); methodology (equal); project administration (equal); resources (equal); software (equal); supervision (equal); validation (equal); visualization (equal); writing – original draft (supporting); writing – review and editing (supporting).

## 7 CONFLICT OF INTEREST

The authors declare that they have no potential conflicts of interest relevant to this article.

8

## Data Availability

Data available on request from the authors.
